# Mechanisms of Time Banditry Behavior on Employees’ Proactive Behavior

**DOI:** 10.3390/bs16050784

**Published:** 2026-05-15

**Authors:** Caiyun Wei, Lanxia Zhang

**Affiliations:** 1 School of Economics and Management, Ningbo University of Technology, Ningbo 315211, China; 2 School of Business Administration, Northeastern University, Shenyang 100167, China; lxzhang@mail.neu.edu.cn

**Keywords:** time banditry behavior, work engagement, work–family conflict, work calling, employees’ proactive behavior

## Abstract

With the development of the internet and communication technology, employee time banditry behavior has become more complex and diversified, with increasing frequency, posing new challenges to enterprise management practices. However, little is known about the effects of such behavior. Based on the conservation of resources theory, this study employed hierarchical regression analysis using data from 474 two-time point questionnaires to explore the relationship between time banditry behaviors and employees’ proactive behaviors, and delved into intermediary mechanisms and boundary conditions. The results show that work engagement and work–family conflict play a chain-mediated role in the effects of time banditry behavior on employees’ proactive behaviors. Work calling moderates the relationship between time banditry behavior and work engagement. The research conclusion enriches the study on the effects of time banditry behavior and provides a new perspective for effectively managing time banditry behavior.

## 1. Introduction

Time is a crucial core resource for employees, being not only valuable and limited but also non-renewable. However, in practice, employees often invest their limited work time in non-work-related activities, relating to what scholars refer to as time banditry behavior. Time banditry behavior involves employees engaging in unauthorized, non-work-related activities during work hours (Martin et al., 2010). It is a ubiquitous and costly issue in organizations (Li et al., 2025) that can have detrimental effects on individuals, teams, and organizations. In recent years, time banditry behavior has gradually drawn scholars’ attention. Nevertheless, the majority of research focuses on its antecedent variables, with insufficient exploration of its effects. While studies suggest that time banditry behavior may reduce employee performance and hinder team building (Bock & Ho, 2009), these conclusions often remain theoretical inferences or are analyzed implicitly within broader concepts. There is limited empirical validation (Harold et al., 2022; Hua & Bao, 2026; Z. Wang et al., 2025) specifically targeting this behavior itself. Systematically examining the influence pathways and effects of time banditry behavior can deepen our understanding of its nature and provide a basis for practical applications. Therefore, it is necessary to employ empirical research methods to investigate the concrete effects of time banditry behavior specifically.

Employees’ proactive behaviors—self-directed, future-focused actions that improve individual and organizational performance (Frese & Fay, 2001)—have gained widespread attention amid increasing organizational uncertainty. Prior research indicates that proactive behavior is influenced by individual factors (e.g., proactive personality, conscientiousness; McCormick et al., 2019; Tu et al., 2020) and situational factors (e.g., leadership, team, job characteristics, organizational factors; Mostafa & EI-Motalib, 2019; Kueny et al., 2019; Yao et al., 2021; Ohly et al., 2006; Malik, 2023; Shi & Gao, 2022; Parker & Collins, 2010). Recent studies increasingly focus on how individuals’ own behaviors shape their attitudes, emotions, and subsequent actions (Johnson et al., 2014; Qin et al., 2018; Liao et al., 2018). Specifically, workplace deviant behaviors reduce work engagement (Bledow et al., 2011) and thereby diminish proactivity (Yalabik et al., 2015). Time banditry behavior, a typical deviant behavior, consumes work resources and may induce guilt and anxiety (Z. Wang et al., 2025), further depleting resources needed for proactive behavior. However, few studies systematically reveal how time banditry behavior influences employees’ proactive behaviors. Given that proactive behavior is critical for individual performance and organizational adaptability (Chen et al., 2021; Raki et al., 2021; Wee & Fehr, 2021), exploring its antecedents has practical implications. Accordingly, this study focuses on the mechanism through which time banditry behavior affects employees’ proactive behavior.

According to the conservation of resources (COR) theory, individuals have limited resources (e.g., time, energy, attention); expending them on non-task activities reduces resources available for task completion (Hobfoll, 1989). This paper argues that time banditry behavior consumes substantial work resources, causing tension and stress that hinder work engagement (Hobfoll, 2011)—a positive state linked to well-being (Christian et al., 2011). Reduced work engagement may lead employees to replenish resources from the family domain to meet work demands, potentially increasing work–family conflict and associated perceived stress (Choi, 2024). To prevent further resource depletion, employees may reduce proactive behaviors. Thus, this study infers that time banditry affects proactive behavior through work engagement and work–family conflict as chain mediators.

Additionally, COR theory notes that individuals are motivated to both conserve existing resources and acquire new ones. Work calling—a source of resource acquisition reflecting profound passion within a domain (Dobrow & Tosti-Kharas, 2011)—can help employees compensate for resource deficits. When time banditry behavior depletes work resources, high work calling provides psychological resources to increase work engagement, thereby reducing work–family conflict and enhancing proactive behavior. Research indicates that abundant resources motivate employees to contribute to the organization (Fletcher et al., 2018). Accordingly, this study proposes that work calling moderates the relationship between time banditry and work engagement.

**Figure 1:**
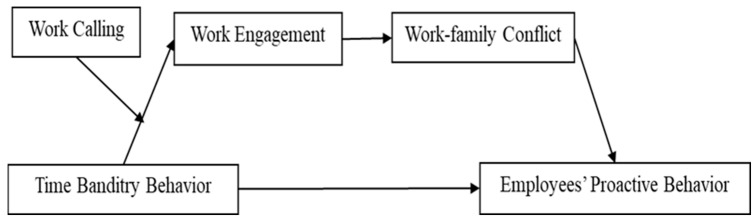
Theoretical model.

In summary, based on COR theory, this study explores the mediating effects of work engagement and work–family conflict, as well as the moderating role of work calling, on how time banditry behavior influences employees’ proactive behavior (Figure 1). It aims to provide a theoretical foundation and empirical evidence for future research on the effects of time banditry behavior.

The theoretical model of this study is shown in Figure 1.

## 2. Theory and Hypotheses

### 2.1. The Impact of Time Banditry Behavior on Work Engagement

According to COR theory, time banditry behavior depletes individuals’ domain-specific resources, leading to resource scarcity that affects employees’ work engagement. Specifically, first, as the boundaries between work time and location become increasingly blurred, employees frequently engage in remote work from home or handle personal matters during work hours. Family obligations often compete with work duties for the same time and attention resources (Hu et al., 2025). Consequently, handling personal matters during work hours—a form of time banditry behavior—consumes employees’ time resources, reducing the time resources available for work and leaving them with insufficient resources to complete work tasks (Lindquist & Kaufman-Scarborough, 2007). Secondly, according to COR theory, when individuals perceive resource loss, they proactively take measures to prevent further depletion (Hobfoll, 1989). When employees consume work resources by browsing non-work-related websites or handling personal emails during work hours, they may adopt defensive mechanisms such as taking additional breaks or reducing work engagement to preserve existing resources (Hobfoll, 1989). Research indicates that individuals’ perceived resource changes can influence subsequent attitudes and behaviors (Halbesleben et al., 2014). Finally, time banditry depletes individual resources, and the depletion of resources is associated with negative emotions such as anxiety. At this point, employees need to mobilize more emotional resources to meet work demands, which further exhausts their emotional reserves. Work engagement refers to individuals fully immersing themselves in tasks (Crawford et al., 2010), requiring sufficient resource support. However, the resource depletion caused by time banditry behaviors reduces the likelihood of employees’ work engagement. Research shows that when employees avoid work, their work engagement decreases (Schaufeli & Taris, 2005).

In summary, this study proposes the following hypothesis:

**Hypothesis** **1.** *Time banditry behavior has a negative impact on work engagement.*

### 2.2. The Mediating Role of Work Engagement

Work engagement is an individual’s positive, sustained, and fulfilling state of involvement in work, exerting positive effects on both personal well-being and role-related performance (Corbeanu & Iliescu, 2023). Based on COR theory, this study posits that work engagement negatively influences work–family conflict. Specifically, first, individuals with high work engagement possess a strong sense of identification, making them more likely to acquire resources such as personal knowledge or skills in the workplace (Demerouti et al., 2001). When individuals accumulate sufficient resources at work, they gain the capacity to manage conflicts between work and family domains, reducing the likelihood of work–family conflict. Second, work–family conflict arises when employees fail to meet the demands or expectations of both their organization and family. When the resources an employee possesses are insufficient to satisfy organizational expectations while also fulfilling family expectations, work and family conflicts inevitably emerge. By fully dedicating themselves to work in the organization, and saving certain resources or avoiding the use of family resources, employees can minimize conflicts between work and family domains. Finally, when employees engage in their work and complete tasks efficiently, they not only gain recognition from leadership and foster harmonious labor–management relations, but also build positive interpersonal relationships with team members. Supportive resources from the organization and positive emotions spilling over into the family domain may enhance employees’ quality of life (Rastogi & Chaudhary, 2018) and reduce work–family conflict. Thus, it is evident that time banditry behaviors consume substantial work resources, preventing employees from fully dedicating themselves to fulfilling job responsibilities. The decline in work engagement further impairs employees’ ability to meet job demands, which may relate to the use of family resources to fulfill work obligations, thereby being associated with work–family conflict.

In summary, this study proposes the following hypothesis:

**Hypothesis** **2.** *Work engagement mediates the relationship between time banditry behavior and work–family conflict.*

### 2.3. The Sequential Mediating Role of Work Engagement and Work–Family Conflict

Based on the above analysis, this study predicts that employees’ time banditry behavior has an indirect impact on employees’ proactive behavior through work engagement and work–family conflict, based on COR. Specifically, time banditry behavior depletes employees’ limited time resources, preventing full commitment to work and hindering timely task completion. Employees may then expend additional time resources or even family time resources to complete their work, relating to work–family conflict. When employees face work–family conflict, they expend substantial resources to mitigate it, relating to resource scarcity. To conserve existing resources, employees may reduce resource-consuming activities, such as proactive behavior, to prevent further resource depletion.

Empirical research supports the detrimental effects of time banditry behavior on work-related outcomes. Hu et al. (2025) showed that time banditry behavior consumes limited work resources, reducing task completion. The resource depletion perspective (Hobfoll, 1989) is further evidenced by Halbesleben et al. (2014), who found that perceived resource changes influence subsequent attitudes and behaviors. Regarding work engagement and work–family conflict, Rastogi and Chaudhary (2018) demonstrated that work engagement enhances quality of life and reduces work–family conflict, while Demerouti et al. (2001) showed that highly engaged employees acquire resources to manage work–family conflicts. Moreover, the sequential mediation logic is consistent with empirical evidence: Karatepe and Olugbade (2016) found that work engagement mediates between work resources and outcomes, with work–family conflict as a subsequent outcome, and Xiao et al. (2018) provided support that workplace deviant behaviors deplete resources, reducing positive behaviors. Collectively, these findings reinforce that work engagement and work–family conflict sequentially mediate the link between time banditry behavior and employees’ proactive behavior.

In summary, this study proposes the following hypothesis:

**Hypothesis** **3.** *Work engagement and work–family conflict play a sequential mediating role in the relationship between time banditry behavior and employees’ proactive behavior.*

### 2.4. The Moderating Role of Work Calling

Work calling refers to a strong passion for one’s job accompanied by a profound sense of meaning and the pursuit of work–personal value alignment (Berg et al., 2010). Higher work calling is associated with better memory of personal values, higher life satisfaction, and engagement in socially beneficial activities (Hagmaier & Abele, 2015). According to COR theory, resources help alleviate scarcity and mitigate negative work demands, but individuals with the same resources may show different engagement levels, suggesting moderating factors. This study argues that work calling moderates the effect of time banditry behavior on work engagement.

Specifically, work calling reflects intrinsic, self-determined motivation (Dobrow et al., 2023). Even when time banditry behavior consumes work resources, high work calling provides autonomous motivations (e.g., intrinsic, identificatory, internalized) that drive work engagement. Moreover, employees with high work calling act consistently with their self-values (French & Domene, 2010), avoiding the concealment of misconduct and engaging in self-reflection and compensatory effort when time banditry behavior occurs. Finally, time banditry behavior reduces autonomy and fails to generate high motivation; employees with high work calling assign greater intrinsic meaning to work and persist under demanding conditions (Bunderson & Thompson, 2009). Work calling also shapes how individuals perceive and respond to resources, offering additional explanatory power (Elangovan et al., 2010).

In summary, this study proposes the following hypothesis:

**Hypothesis** **4.** *Work calling negatively moderates the relationship between time banditry behavior and work engagement. The higher the work calling, the weaker the impact of time banditry behavior on work engagement, and vice versa.*

Based on Hypotheses 2 and 4, this study argues that work calling moderates the mediating role of work engagement between time banditry behavior and work–family conflict. Specifically, high work calling strengthens employees’ sense of work responsibility and moral obligation (Mawritz et al., 2014), enabling them to actively complete tasks and thereby reduce the transmission effect of work engagement. In contrast, low work calling provides fewer psychological resources and internal constraints (Bowling et al., 2011). When time banditry behavior occurs, employees with low work calling cannot sustain work engagement and may replenish resources from the family domain to meet work demands, leading to work–family conflict.

In summary, this study proposes the following hypothesis:

**Hypothesis** **5.** *Work calling negatively moderates the mediating effect of work engagement in the relationship between time banditry behavior and work–family conflict. The higher the work calling, the weaker the indirect effect of time banditry behavior on work–family conflict through work engagement, and vice versa.*

Based on Hypotheses 3 and 4, this study argues that work calling moderates the serial mediating effect of work engagement and work–family conflict in the relationship between time banditry behavior and employees’ proactive behavior. For employees with high work calling, when time banditry behavior reduces work engagement, their higher psychological resources and autonomous motivation help them restrain time banditry behavior, conserve resources for work, and complete tasks during work hours. This not only provides a sense of accomplishment but also allows them to approach family responsibilities with fuller emotional and resource reserves, thereby reducing work–family conflict and preserving resources for proactive behavior. Conversely, for employees with low work calling, time banditry behavior may serve as an escape from work, and they are unlikely to reduce such behavior even when it diminishes work engagement and efficiency. To avoid punishment for poor performance, they may obtain resources from the family domain to complete work tasks, leading to work–family conflict due to depleted family resources. Resolving this conflict consumes significant resources, thereby reducing the resources available for proactive behavior and diminishing its likelihood.

In summary, this study proposes the following hypothesis:

**Hypothesis** **6.** *Work calling negatively moderates the mediating effect of work engagement and work–family conflict in the relationship between time banditry behavior and employees’ proactive behavior. The higher the work calling, the weaker the indirect effect of time banditry behavior on employees’ proactive behavior through work engagement and work–family conflict, and vice versa.*

## 3. Materials and Method

### 3.1. Sample and Procedure

This study collected data through a questionnaire survey method, with the questionnaires distributed via third-party distribution. Before the survey, researchers designed the questionnaires using “Question Star” to generate electronic links and QR codes. Subsequently, they contacted alumni networks and relevant corporate executives to explain the purpose and methods of the survey, securing their support and compiling a list of participating companies. Next, the researchers coordinated with the HR department heads of these companies to facilitate questionnaire distribution and retrieval. Since this survey employed a two-time point measurement approach, the HR departments were required to provide employee lists for the survey prior to distribution, enabling researchers to assign identifiers (e.g., A1-1 for the first employee in the first company) to facilitate the matching of responses from both timepoints. Researchers sent the first and second sections of the questionnaire to HR contacts via electronic links and QR codes at Time point 1 and 6 weeks later (Time point 2), respectively, instructing them to forward the materials to designated employees and remind them to complete the surveys. The 6-week interval was chosen because Podsakoff et al. (2003) indicated that this duration effectively mitigates common method bias, as shorter intervals would otherwise increase correlations between variables.

Researchers collected data via the “Question Star” platform and excluded invalid questionnaires with unusually short completion times or responses showing obvious response patterns. To encourage and thank participants, the researchers established a cash reward mechanism ranging form 3 to 5 yuan, where each participant has a 20% chance of winning. Time point 1 primarily measured time banditry behavior and work calling; Time point 2 focused on work engagement, work–family conflict, and employees’ proactive behavior. At Time point 1, 595 questionnaires were collected, yielding 547 valid responses after removing those with excessively short completion times or patterned answers, resulting in an effective recovery rate of 91.9%. At Time point 2, 504 questionnaires were collected, with 474 valid responses after invalid removals, achieving an effective recovery rate of 79.7%. A total of 474 valid matched questionnaires were collected across both time points, with an overall effective recovery rate of 79.66%. The basic characteristics of the sample are presented in Table 1.

### 3.2. Variable Measurement

Variables were measured using a five-point Likert scale, where 1 indicates “completely disagree”, 2 “mostly disagree”, 3 “uncertain”, 4 “mostly agree”, and 5 “completely agree”.

Time banditry behavior was measured using eight items adapted by the authors (see Appendix A). We first integrated the items from the “Time Theft” (Harold et al., 2022) and “Time Banditry Questionnaire” (Brock et al., 2013) scales, removing items that were similar or contextually inconsistent. The remaining items formed a new scale. Subsequently, we administered two waves of questionnaire surveys (the first for exploratory factor analysis, and the second for confirmatory factor analysis as well as reliability and validity testing) to systematically examine the reliability and validity (including content validity, construct validity, and discriminant validity) of the new scale. Based on the results, we finalized the items of the formal scale. With representative items such as “At work, I browse news or unrelated webpages”, Cronbach’s α is 0.846.

Work engagement: A one-dimensional six-item measurement scale developed by Rich (Rich et al., 2010) was adopted, with representative items such as “I am fully concentrated at work”; Cronbach’s α is 0.923.

Work calling was measured using four items from the Mission and Career Questionnaire (CVQ) developed by Dik and Duffy (2009). with representative items such as “My job helps me achieve my life goals”; Cronbach’s α is 0.904.

Work–family conflict: The measurement scale developed by Netemeyer et al. (Netemeyer et al., 1996) was adopted, which includes five items, with representative examples such as “Due to work taking up a significant amount of my time, it makes it difficult for me to fulfill my family responsibilities”; Cronbach’s α is 0.932.

Employees’ proactive behavior: A measurement scale developed by Griffin et al. (Griffin et al., 2007) was used, which includes three items, with representative items such as “I will develop better methods to accomplish my important work”; Cronbach’s α is 0.877.

### 3.3. Date Analysis Procedure

Data analysis was performed using SPSS 25.0, AMOS 21.0, PROCESS 4.1. First, we performed AMOS 21.0 to conduct confirmatory factor analysis (CFA). Second, we performed Harman’s single-factor test (SPSS 25.0) and latent method factor (AMOS 21.0) to check for a common method bias. Third, we performed SPSS 25.0 to calculate descriptive statistics and Pearson’s correlation coefficients to examine the relationships among the variables. Fourth, to rigorously test the mediation and moderated mediation hypotheses, we employed the PROCESS macro in SPSS. The bias-corrected Bootstrap method with 5000 resamples was applied to estimate confidence intervals (CIs) for significance testing.

## 4. Results

### 4.1. Confirmatory Factor Analysis

This study uses AMOS 21.0 to conduct confirmatory factor analysis (CFA) to test the discriminability between the five constructs in the formal questionnaire: time banditry behavior, work engagement, work–family conflict, work calling, and employees’ proactive behavior. The discriminant validity of each variable is tested by comparing the fitting indicators of different factor models, as shown in Table 2. As shown in Table 2, the fitting indicators of the five-factor model (χ^2^/df = 2.483, TLI = 0.943, CFI = 0.950, RMSEA = 0.056) are significantly better than the other four models, indicating good discriminant validity among the five main variables involved in this chapter.

### 4.2. Common Method Bias

Although this article collected data at two time points, there may be common methodological biases in the survey data as all variables involved were self-evaluated by employees. Therefore, this article uses Harman’s single-factor common method bias test to test it. The results showed that the first factor accumulated 34.493% variance, which is less than 40% of the judgment criteria. It can be seen that the common method bias problem of the data in this article is not serious. In addition, in order to further prove the conclusion of the data in this article, the latent method factor was used to re-examine the common method bias. The results showed that the model incorporating common method factors (χ^2^/df = 2.040, TLI = 0.960, CFI = 0.968, RMSEA = 0.047) did not show significant improvement (ΔTLI = 0.017, ΔCFI = 0.018, ΔRMSEA = 0.009) compared to the baseline model, once again proving that there is no serious common method bias problem in this paper.

### 4.3. Descriptive Statistical Analysis

SPSS 25.0 was used to measure the means, standard deviations, and correlation coefficients between variables, as shown in Table 3. According to Table 3, there is a significant negative correlation between time banditry behavior and work engagement (r = −0.361, *p* < 0.001) and employees’ proactive behavior (r = −0.236, *p* < 0.001). There is a significant negative correlation between work engagement and work–family conflict (r = −0.235, *p* < 0.001). There is a significant negative correlation between work–family conflict and employees’ proactive behavior (r = −0.256, *p* < 0.001), and this hypothesis has been preliminarily validated.

### 4.4. Hypothesis Testing

**Figure 2:**
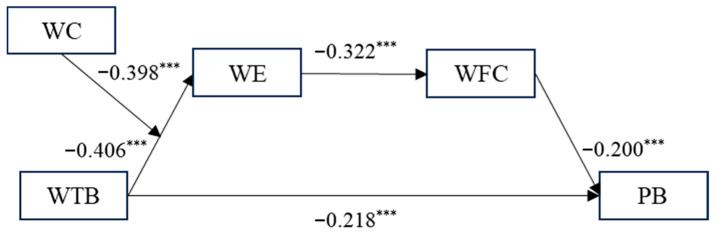
Model path coefficient. *** *p* < 0.001.

This article first uses SPSS 25.0 to perform hierarchical regression analysis on the samples, and the test results are shown in Table 4. We also test the model using structural equation modeling, and the test results are shown in Figure 2.

According to M2 in Table 4, after controlling for gender, age, educational background, marital status, and nature of enterprise, time banditry behavior has a significant negative impact on work engagement (β = −0.340, *p* < 0.001). Therefore, hypothesis 1 is validated. According to M7 in Table 4, after controlling for gender, age, educational background, marital status, and nature of enterprise, and incorporating both time banditry behavior and work engagement, work engagement has a significant negative impact on work–family conflict (β = −0.174, *p* < 0.001). Therefore, hypothesis 2 is validated. According to M11 in Table 4, after controlling for gender, age, educational background, marital status, and nature of enterprise, the addition of time banditry behavior, work engagement, and work–family conflict, work engagement (β = 0.612, *p* < 0.001) and work–family conflict (β = −0.119, *p* < 0.01) have a significant impact on employees’ proactive behavior. Therefore, hypothesis 3 is validated. According to M3 in Table 4, after controlling for gender, age, educational background, marital status, and nature of enterprise, the interaction terms of time banditry behavior, work calling, and time banditry behavior and work calling were also included. The interaction term between time banditry behavior and work calling had a significant negative impact on work engagement (β = −0.393, *p* < 0.01). Therefore, hypothesis 4 was validated. Figure 2 shows that the results of the structural equation modeling (χ^2^/df = 2.215, TLI = 0.990, CFI = 0.998, RMSEA = 0.051) are consistent with those of the hierarchical regression analysis.

In order to further test the mediating effect of work engagement, we conducted a Bootstrap analysis using the PROCESS macro for SPSS 25.0. By conducting 5000 repeated extractions of valid samples and selecting the bias corrected non-parametric percentile method, the results are shown in Table 5.

As shown in Table 5, the indirect effect of work engagement on the relationship between time banditry behavior and work–family conflict is 0.0749, with a 95% confidence interval of [0.0286, 0.1193], excluding zero, indicating a significant mediating effect of work engagement. Therefore, hypothesis 2 is validated; the indirect effect of work engagement and work–family conflict on the relationship between time banditry behavior and employees’ proactive behavior is −0.0061, with a 95% confidence interval of [−0.0126, −0.0010], excluding zero, indicating a significant continuous mediating effect of work engagement and work–family conflict. Therefore, hypothesis 3 is validated.

**Figure 3:**
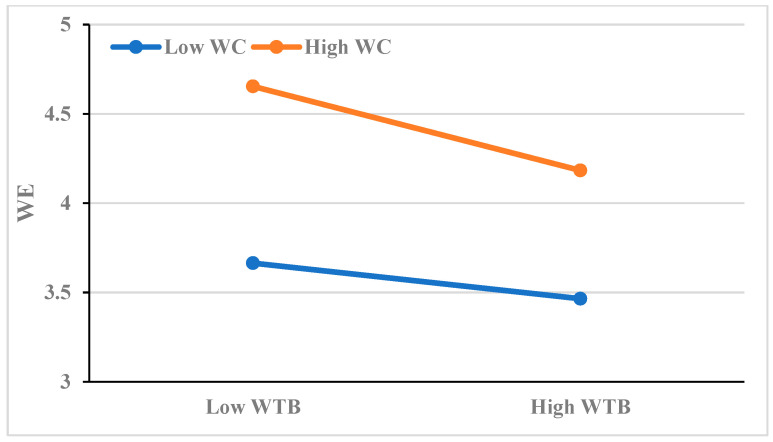
Plot of the moderating influence of work calling on the relationship between time banditry behavior and work engagement.

To further clarify the direction and trend of the moderation effect, using one standard deviation above and below the mean as the standard, work calling is divided into high and low levels, and a regression effect graph of time banditry behavior on work engagement is established (see Figure 3). From Figure 3, it can be seen that when work calling is high, the negative effect of time banditry behavior on work engagement weakens. When work calling is low, the negative effect of time banditry behavior on work engagement increases. Thus, hypothesis 4 is once again verified.

This article uses the Process program in SPSS 25.0 analysis tool and employs the Bootstrap method to repeat sampling 5000 times to test the moderated mediation effect. The results are shown in Table 6. According to Table 6, when work calling is below one standard deviation, the indirect effect value of work engagement on time banditry behavior and work–family conflict is 0.0282, with a 95% confidence interval of [−0.0066, 0.0681]. When work calling is higher than one standard deviation, the indirect effect value of work engagement on the relationship between time banditry behavior and work–family conflict is 0.0631, with a 95% confidence interval of [0.0193, 0.1125]. The mediating effect value between the two groups is −0.0806, with a 95% confidence interval of [−0.1303, −0.0309], excluding zero. The index of moderated mediation is 0.0200, with a 95% confidence interval of [−0.0083, 0.0574], including zero. Hypothesis 5 is not verified.

When work calling is below one standard deviation, the indirect effect value of work engagement and work–family conflict between time banditry behavior and employees’ proactive behavior is −0.0022, with a 95% confidence interval of [−0.0064, 0.0007]. When work calling is higher than one standard deviation, the indirect effect value of work engagement and work–family conflict between work time banditry behavior and employees’ proactive behavior is −0.0051, with a 95% confidence interval of [−0.0113, −0.0009]. The mediating effect of the two is −0.0144, with a 95% confidence interval of [−0.0315, −0.0026], excluding zero. The index of moderated mediation is −0.0016, with a 95% confidence interval of [−0.0052, 0.0006], including zero. Hypothesis 6 is not verified.

## 5. Discussion and Conclusions

This article, based on COR theory, uses 474 two-time point questionnaire surveys and employs hierarchical regression analysis to explore a moderated mediation model of the impact of time banditry behavior on employees’ proactive behavior, with work engagement and work–family conflict as mediating variables and work calling as a moderating variable, verifying relevant hypotheses.

### 5.1. Research Conclusions

In conclusion, time banditry behavior depletes employees’ limited work resources, thereby exerting a negative effect on work engagement. Work engagement, in turn, reduces work–family conflict by enabling employees to acquire resources that facilitate cross-domain management. Moreover, work engagement mediates the relationship between time banditry behavior and work–family conflict. Notably, work engagement and work–family conflict function as sequential mediators in the link between time banditry behavior and employees’ proactive behavior: time banditry behavior diminishes work engagement, which hampers task completion and compels employees to draw on family resources, thereby intensifying work–family conflict and further depleting resources, ultimately reducing proactive behavior. Additionally, work calling moderates the negative impact of time banditry behavior on work engagement, such that employees with high work calling compensate for resource loss through self-reflection and increased work investment.

However, the moderating effect of work calling on the mediation of work engagement between time banditry and work–family conflict is not significant. Similarly, work calling does not significantly moderate the serial mediation of work engagement and work–family conflict in the relationship between time banditry and employees’ proactive behavior. A recent study suggests that employees’ time banditry may be driven not only by self-oriented motives but also by well-intentioned other-oriented and work-oriented motives (Hu et al., 2025). Other-oriented motives refer to helping family or friends with (potential) issues, even without being asked; work-oriented motives often function by reducing strain or replenishing energy. Accordingly, time banditry might sometimes increase work engagement and reduce work–family conflict. This contrasts with our findings that time banditry reduces engagement and increases conflict, which may explain why the moderated mediation effects were not verified.

### 5.2. Theoretical Contributions

Firstly, explore the effect of time banditry behavior on employees’ proactive behavior. Previous studies on time banditry behavior have mostly focused on its influencing factors (Li et al., 2025; S. Zhao & Ma, 2023), with few studies examining the effects of time banditry behavior. This study explores the impact of time banditry behavior on employees’ proactive behavior from the perspective of actors, and delves into the underlying mechanism and boundary conditions, providing a theoretical framework and empirical evidence for revealing the negative impact mechanism of time banditry behavior on actors themselves. Additionally, this study responds to the academic call for further exploration of its outcomes (Harold et al., 2022).

Secondly, this study expands the scope of time banditry behavior to the family realm. Previous studies have focused on the factors and effects of time banditry behavior in the workplace, with little extension to the family domain. However, research has shown that time banditry behavior may be driven by prosocial motives, manifested as proactively helping their family or friends solve (potential) issues without being asked (Hu et al., 2025). This suggests a connection between time banditry behavior and the family domain. This article explores the mediating role of work engagement and work–family conflict in time banditry behavior and employees’ proactive behavior. Exploring the mediating role of work–family conflict between time banditry behavior and employees’ proactive behavior in the family domain not only fills the gap in previous research that only focused on time banditry behavior in the work domain, but also enriches the research results of time banditry behavior.

Finally, the study explored the moderating effect of work calling from the perspective of individual differences. The research results found that for employees with a high work calling, time banditry behavior has a reduced impact on work engagement. For employees with low work calling, time banditry behavior has an enhanced impact on work engagement. This research conclusion confirms the moderating effect of work calling, providing inspiration for alleviating the negative effects of time banditry behavior and enriching the application of calling research to a certain extent. However, the moderating effect of work calling on mediation (work engagement) and serial mediation (work engagement, work–family conflict) was not significant. A plausible explanation is that time banditry behavior can also be driven by other-oriented or work-oriented motives (e.g., reducing strain or replenishing energy), which may occasionally increase engagement and reduce conflict (Hu et al., 2025). This contrasts with our main findings and suggests that the motivational heterogeneity of time banditry may offset the buffering role of work calling. Future research should further disentangle these competing mechanisms.

### 5.3. Managerial Implications

Firstly, managers should pay attention to employees’ time banditry behavior. Research has shown that time banditry behavior suppresses employees’ proactive behavior through work engagement and work–family conflict, and has a negative impact on the company. Therefore, managers should not ignore the negative impact of time banditry on the enterprise, and measures should be taken to reduce the probability of employees engaging in time banditry. Existing research has shown that perceived overqualification (S. Zhao & Ma, 2023), relationship conflict (P. Zhao et al., 2025), and psychological distress (Alajhar et al., 2025) can all lead to time banditry behavior. Managers could emphasize collectivism and solidarity, creating an atmosphere conducive to collegiality, or offering training on empathy and mindfulness (Hanley & Garland, 2019). Additionally, managers should assign work tasks to employees that match their qualifications to avoid perceived overqualification and psychological distress that can lead to time banditry behavior.

Secondly, managers should pay attention to employees’ work engagement and work–family conflict. Research has shown that time banditry behavior can have a negative impact on employees’ proactive behavior through work engagement and work–family conflict. Therefore, managers should strive to create a comfortable working environment and incentive systems to stimulate employees’ work engagement, implement family–friendly policies, provide support to employees, enable them to complete their work during working hours, and reduce the drain on family resources, thereby increasing their family engagement and reducing work–family conflicts.

Finally, managers should pay attention to employees’ work calling. Research has shown that the negative impact of time banditry behavior on work engagement is moderated by work calling. Therefore, in talent selection, managers can consider work calling as one of the factors and intentionally choose people who have work calling. Work calling can be cultivated or enhanced in practical work, so in daily management work, managers should deliberately create an environment that can stimulate and protect employees’ work calling. In addition, managers should also pay attention to training employees’ work calling, by strengthening the training of employees’ work calling, so that they can complete work tasks well in any situation.

### 5.4. Limitations and Future Research Directions

Although most of the hypotheses proposed in this study have been confirmed, there are still shortcomings that need to be improved.

Firstly, this study utilizes data collected at two time points to validate the hypothesis, with all variables being self-reported by participants. Although self-reporting is often considered optimal in organizational research, participants may feel pressured to provide socially desirable responses (C. Wang et al., 2025). To examine common method bias, this study conducted Harman’s single-factor test and further employed latent method factor analysis for verification. The results indicate that although the issue of common method bias is not severe, it cannot be completely ruled out. Future research can attempt to collect data at multiple time points while using a combination of employee self-evaluation and peer evaluation, situational experimental methods, and other more objective methods to measure variables.

Secondly, all variables in this study are individual-level variables, which to some extent cannot fully explain the impact mechanism of time banditry behavior on employees’ proactive behavior. Future research could incorporate team-level or organizational-level variables and explore the relationship between time banditry behavior and employees’ proactive behavior in a cross-layer manner.

## Figures and Tables

**Table 1:** Demographic characteristics of the sample.

Variable	Items	Count	%	Cum.%
Gender	Male	214	45.1%	45.1%
	Female	260	54.9%	100%
Age	18–30	215	45.4%	45.4%
	31–40	204	43%	88.4%
	41–50	31	6.5%	94.9%
	≥51	24	5.1%	100%
Educational background	Associate degree and below	55	11.6%	11.6%
	Bachelor’s degree	247	52.1%	63.7%
	Undergraduate and above	172	36.3%	100%
Marital Status	Unmarried	196	41.4%	41.4%
	Married	273	57.5%	98.9%
	Divorced	5	1.1%	100%
Nature of enterprise	State-owned	141	29.7%	29.7%
	Private	196	41.4%	71.1%
	Foreign-invested enterprise	20	4.2%	75.3%
	Civil servant/Public institution employee	117	24.7%	100%

Note: N = 474.

**Table 2:** Results of confirmatory factor analysis.

	χ^2^	df	χ^2^/df	CFI	TLI	RMSEA
WTB, WE, WFC, WC, PB	715.201	288	2.483	0.950	0.943	0.056
WTB, WE + PB, WFC, WC	1148.325	293	3.919	0.899	0.888	0.079
WTB, WE + WC, WFC, PB	1424.044	293	4.860	0.867	0.852	0.090
WTB, WE, WFC + WC, PB	2168.282	293	7.400	0.779	0.755	0.116
WTB, WE + WFC + WC, PB	3232.280	296	10.920	0.655	0.621	0.145
WTB + WE + WFC + WC, PB	4514.197	298	15.148	0.504	0.459	0.173
WTB + WE + WFC + WC + PB	4855.784	299	16.240	0.464	0.417	0.179

Notes: N = 474; WTB = time banditry behavior; WE = work engagement; WFC = work–family conflict; WC = work calling; PB = employees’ proactive behavior.

**Table 3:** Means, standard deviations and correlations.

	1	2	3	4	5
WTB	1				
WE	−0.361 ***	1			
WFC	0.219 ***	−0.235 ***	1		
WC	−0.295 ***	0.595 ***	−0.159 ***	1	
PB	−0.236 ***	0.644 ***	−0.256 ***	0.488 ***	1
M	2.637	4.012	2.703	3.847	4.021
SD	0.920	0.772	1.064	0.914	0.768

Notes: *** *p* < 0.001.

**Table 4:** Linear regression analysis model.

Variable	WE			WFC				PB			
	M1	M2	M3	M4	M5	M6	M7	M8	M9	M10	M11
Gender	−0.036	−0.033	−0.006	−0.124 **	−0.126 **	−0.132 **	−0.132 **	−0.059	−0.057	−0.090 *	−0.051
Age	0.031	−0.010	0.013	−0.049	−0.036	−0.043	−0.027	−0.034	−0.061	−0.046	−0.057
Educational background	−0.001	0.028	0.074	0.064	0.048	0.064	0.053	0.003	0.022	0.019	0.011
Marital status	0.194 ***	0.169 ***	0.056	−0.079	−0.064	−0.036	−0.035	0.197 ***	0.180 **	0.177 **	0.069
Nature of enterprise	−0.010	0.001	−0.034	0.029	0.023	0.027	0.023	0.007	0.015	0.015	0.017
WTB		−0.340 *** (−361 ***)	0.153 (0.154)		0.197 *** (0.219 ***)		0.138 ** (0.154 ***)		−0.222 *** (−0.236 ***)		0.009 (0.013)
WE						−0.221 *** (−0.235 ***)	−0.174 *** (−0.180 **)				0.612 *** (0.622 ***)
WFC										−0.252 *** (−0.256 ***)	−0.119 ** (−0.112 **)
WC			0.800 *** (0.807 ***)								
WTB × WC			−0.393 ** (−0.398 **)								
R^2^	0.046	0.156 (0.130)	0.412 (0.405)	0.039	0.076 (0.048)	0.085 (0.055)	0.101 (0.076)	0.034	0.081 (0.055)	0.095 (0.065)	0.432 (0.426)
F	4.503 ***	14.360 *** (70.595 ***)	40.775 *** (106.463 ***)	3.759 **	6.361 *** (23.755 ***)	7.252 *** (27.640 ***)	7.497 *** (19.365 ***)	3.336 **	6.888 *** (27.733 ***)	8.198 *** (32.987 ***)	44.247 *** (116.292 ***)

Notes: * *p* < 0.05; ** *p* < 0.01; *** *p* < 0.001. The values in parentheses are the analysis results without control variables.

**Table 5:** Bootstrap test results of mediation effects.

Path	Effects	SE	95%CI	
			Lower	Upper
WTB → WE → WFC	0.0749	0.0231	0.0286	0.1193
WTB → WE → WFC → PB	−0.0061	0.0030	−0.0126	−0.0010

**Table 6:** Bootstrap test results of moderated mediation effects.

Path	Moderator Variable	Effects	SE	95%CI	
				Lower	Upper
WTB → WE → WFC	Low WC	0.0282	0.0190	−0.0066	0.0681
	High WC	0.0631	0.0234	0.0193	0.1125
	The difference	−0.0806	0.0253	−0.1303	−0.0309
WTB → WE → WFC → PB	Low WC	−0.0022	0.0018	−0.0064	0.0007
	High WC	−0.0051	0.0027	−0.0113	−0.0009
	The difference	−0.0144	0.0076	−0.0315	−0.0026

## Data Availability

The raw data supporting the conclusions of this article will be made available by the authors.

## Appendix A

**Table A1:** Items of time banditry behavior.

N	Items
1	I read books unrelated to my job at work
2	I sometimes watch entertainment programs at work
3	I sometimes play games at work
4	I sometimes play with my phone at work
5	I often shop online at work
6	I browse news or unrelated webpages at work
7	I often chat with my colleagues about topics unrelated to work
8	I will answer personal phone calls at work
